# Understanding and living with glaucoma and non-communicable diseases like hypertension and diabetes in the Jhaukhel-Duwakot Health Demographic Surveillance Site: a qualitative study from Nepal

**DOI:** 10.3402/gha.v7.25358

**Published:** 2014-10-20

**Authors:** Suraj Shakya-Vaidya, Lene Povlsen, Binjwala Shrestha, Andrej M. Grjibovski, Alexandra Krettek

**Affiliations:** 1 Department of Internal Medicine and Clinical Nutrition, Institute of Medicine, Sahlgrenska Academy, University of Gothenburg, Gothenburg, Sweden; 2 Nordic School of Public Health NHV, Gothenburg, Sweden; 3 Department of Community Medicine and Public Health, Institute of Medicine, Tribhuvan University, Maharajgunj, Kathmandu, Nepal; 4 Department of International Public Health, Norwegian Institute of Public Health, Oslo, Norway

**Keywords:** barriers to health care, diabetes, health literacy, hypertension, non-communicable disease, primary open-angle glaucoma

## Abstract

**Background:**

Primary open-angle glaucoma (POAG) is one of the most common causes of irreversible blindness. A possible association between POAG and non-communicable diseases such as hypertension and diabetes suggests that the incidence of POAG may increase. People with POAG in Nepal usually present late to hospital and have poor knowledge of glaucoma.

**Objectives:**

Anticipating a knowledge gap regarding these diseases, this study aimed to explore the knowledge of POAG, hypertension, and diabetes in the community and barriers to health care.

**Design:**

We conducted this qualitative study in the Jhaukhel-Duwakot Health Demographic Surveillance Site (JD-HDSS), a peri-urban community near Kathmandu, a capital city of Nepal. To study how disease influences knowledge, we conducted focus group discussions separately for men and women with and without pre-existing POAG, hypertension, and diabetes. Data were analyzed using the framework analysis approach.

**Results:**

Although people suffering from POAG, hypertension, and/or diabetes exhibited adequate knowledge of hypertension and diabetes, they lacked in-depth knowledge of POAG. People believed mostly in internal health locus of control. Perception of disease consequences and impact of disease on daily life was influenced by pre-existing POAG, hypertension, and/or diabetes but only in men. Gender disparity was observed regarding health literacy, health perception, and health barriers, which put women in a more difficult situation to tackle their health. We also revealed a gap between knowledge, attitude, and practice of health among women and healthy men.

**Conclusion:**

Although people in JD-HDSS exhibited adequate knowledge regarding hypertension and diabetes, they lacked in-depth knowledge about POAG. This study demonstrated gender difference in health literacy and access to health care, making women more vulnerable towards disease. We also demonstrated a gap between knowledge, attitude, and practice of health. However, tailored health literacy programs may bring changes in the health status in the community.

Worldwide, glaucoma is one of the most common causes of blindness, outnumbered only by cataract (1). Primary open-angle glaucoma (POAG) occurs frequently in most countries, including Nepal (2, 3), and is the most common cause of irreversible blindness (1). In addition to age, gender, and family history of glaucoma, reports suggest that systemic hypertension and diabetes mellitus (diabetes) are risk factors for POAG (4–6). Earlier we demonstrated possible association between POAG, hypertension, and diabetes (7). Moreover, Nepal is experiencing increased incidence of non-communicable diseases (NCDs) including hypertension and diabetes (8–10), implying the likelihood that POAG may become more prevalent.

The current prevalence of POAG in Nepal is 1.24% (3). Our recently published hospital-based study demonstrates that based on the total number of different ethnic groups attending hospitals, 6.2% Gurungs, 3.5% each of Newars and Brahmin, and 3.2% Tharus were diagnosed with POAG for the first time (7). The above study also reveals moderate to severe visual impairment in 9.8% of POAG cases at time of diagnosis and 4.7% legally blind due to glaucoma (7). Another study from Nepal demonstrates severe loss of visual field among glaucoma patients who present late to hospital (11). Additionally, another study originating from Nepal reports lack of sufficient knowledge of glaucoma among patients who attend eye hospitals (12). A study from the United States shows that, people who know that they are at risk of glaucoma fail to return for follow-up visits even when they receive free health care (13), suggesting that disease detection depends on health-seeking behavior.

Health-seeking behavior is described by theories and concepts such as the health belief model (HBM) (14), health locus of control (15), and health literacy (16). According to the HBM, perceived severity of and vulnerability to a disease, as well as perceived benefits and barriers to changing health behavior, will decide an individual's attitude and decisions about the disease (14). Health locus of control refers to the extent to which individuals believe they participate in and control events that affect their health. Individuals with an internal locus of control believe that their own behavior and active involvement in health care is vital to improving their outcome (15). Low health literacy associates with poorer health outcomes, less learning from health education, less use of health services, and less involvement in self-care and self-management of health problems (16). Therefore, low health literacy, an implementation gap between knowledge and practice, and/or barriers to seeking health care might explain why POAG patients present to hospitals late in their disease. The present study aimed to explore knowledge regarding POAG, hypertension, and diabetes in the Jhaukhel-Duwakot Health Demographic Surveillance Site (JD-HDSS), a peri-urban community located near Kathmandu, the capital city of Nepal. We also aimed to identify perceptions of and potential barriers to lifestyle changes and seeking health care.

## Methods

We conducted a qualitative study using focus group discussions (FGDs). This approach is particularly recommended for exploring experiences and perceptions from individuals’ own perspectives (17).

### Study setting

The study was carried out during December 2013 in the JD-HDSS in Nepal's Bhaktapur district, about 13 kilometers from Kathmandu (18). FGDs were conducted in an open and quiet room in the office of a local political party, which was known to all participants and located close to their homes.

### Participants

To explore knowledge regarding POAG, hypertension, and/or diabetes, we enrolled male and female participants, aged 25–45 years old, from various occupations (e.g. housewife, student, farmer, businessman, and teacher). We chose this particular age group because earlier studies from Nepal demonstrate hypertension and diabetes even in younger individuals who are <40 years of age (9, 10). Because we aimed to explore whether pre-existing diagnoses influence knowledge of POAG, hypertension, and diabetes, we included people both with and without these diseases.

Two female community health volunteers (FCHVs) helped recruit FGD participants. Households with potential participants were identified partly from the JD-HDSS database and partly by the FCHVs, because FCHVs had gained familiarity with every household in the community during frequent visits to collect data for JD-HDSS surveys. FCHVs visited households to inform about the study and ask members if they were interested in participating in the FGDs. Participants of FGD self-reported the disease which was not clinically reconfirmed but was supported by the fact that they were on medication for the same disease. Interested household members (32 men and 35 women) who fulfilled the criteria were enrolled in the study.

After enrollment, the researchers carefully evaluated the list of 67 participants. We distributed the men into four groups: two for those affected by disease (one group with eight and another with seven participants), and two groups for those unaffected by disease (nine and eight participants). We also divided the women into four groups: two for those affected by disease (nine and eight participants), and two for those unaffected by disease (nine participants each).

### Data collection

To develop an FGD guide, SSV, LP, and AK discussed content and reviewed relevant literature (19, 20). The guide was then developed using open-ended core questions covering areas such as perceptions of health; knowledge of POAG, hypertension, and/or diabetes; change of lifestyle; and access to health care. When required, we asked probing questions to ensure that all issues were addressed and understood correctly.

SSV and BS – PhD students who are fluent in Nepalese – conducted eight FGDs in Nepali language. Each FGD lasted approximately 60 minutes. To pre-test the FGD guide, BS moderated the first FGD and SSV served as note taker. Because the pre-test did not require any major corrections, we included it in the study. SSV moderated all subsequent FGDs and BS served as note taker.

The moderator began each session by greeting group members and thanking them for their participation. After introducing the research team and explaining the purpose of the FGD, the moderator asked participants to introduce themselves to the group. When participants appeared comfortable, the moderator started the FGD by asking an open-ended question about health in general. This was then followed by the question ‘Have you heard about non-communicable diseases? Please share your knowledge and experience about such diseases with your friends in this group’. Thereafter, we inquired, ‘Have you heard about a disease called glaucoma? Can you discuss what you know about this disease with the group?’ The moderator encouraged quiet participants to speak by asking questions like, ‘What is your opinion?’ and ‘Would you like to share something with us?’

We used a digital tape recorder to document all FGDs. Additionally, the note taker recorded information about the dynamics of the group (e.g. verbal and non-verbal cues, body language, and how and with whom participants interacted). Audio taped data were stored in an external hard drive. The external hard drive and notes were securely kept by the researcher and no access was given to anyone outside the research group.

### Data analysis

We used framework analysis that is used for health research to explore specific information and possibly provide recommendations for changes in health care (21–23) . Although the general approach in framework analysis is inductive, this method also provides flexibility to use both predetermined and potentially emerging themes (23).

Our analysis followed seven steps (21, 23):

1. A community nurse with previous experience in transcribing qualitative data transcribed all audio data verbatim in Nepali. Next, an experienced information and technology graduate translated the transcripts into English. To ensure data reliability, the first and third authors reviewed both the transcribed and translated data to verify that transcriptions and translations were done correctly. Only a few corrections were made in the translated document after a consensus between both authors.
2. We read the transcripts several times to familiarize ourselves with the data.
3. Next, we identified themes using predetermined and emerging issues identified during the familiarization.
4. Specific pieces of relevant data were coded with textual codes.
5. Finally, we constructed a working thematic framework by grouping codes with similar content into categories, and grouping categories with similar concepts into themes.
6. Next, data were charted into framework matrix to enable us to read across the entire data set.
7. Likewise, to help interpret data we did data mapping exposing similarities and differences between various FGDs.

The analysis process was carried out manually by listing, tabulating, and mapping the data in Word 2013. SSV and BS conducted the analysis. SSV has worked as clinician and researcher and participated in earlier community-based health research studies in Nepal, including FGDs to evaluate health services. BS (public health sociologist) participated in qualitative research studies in Nepal while pursuing her Master's degree in Community Medicine and Public Health.

### Ethical consideration

This study conforms to the declaration of Helsinki for research involving humans and was approved by the Nepal Health Research Council. Verbal informed consent was read out to all FGD participants and each participant gave verbal consent to participate in the study, including the use of tape recorders, and notes. We emphasized that personal data would be stored securely and not shared with anyone outside the research group, and that personal identifiers would be removed or disguised prior to publication. We also informed all participants that they could withdraw from the FGD at any time.

## Results

We enrolled 67 respondents and none declined to participate or dropped out during the study. Out of 67, 32 were men with 15 having pre-existing disease (POAG, hypertension and/or diabetes) and 17 were healthy. Thirty-five were women with 17 having above diseases and 18 were without such diseases. The youngest man who suffered from disease was 37 years old and the remaining 14 male participants were ≥40 years. In contrast, out of 17 women with disease, four were <40 years and the youngest was 30 years old. Healthy participants were distributed equally in all age groups. Participants belonged to Newar, Brahmin, and Chettri ethnic groups.

The results from the FGDs are presented in four themes related to the main aspects covered in the FGDs as shown in Table 1.

**Table 1:** Main themes and categories in focus group discussions

Themes	Categories
Health and health beliefs	Perceptions of health Perception of cause of disease
Knowledge of diseases and their prevention	Hypertension and diabetes Glaucoma Preventive measures
Perceptions of diseases	Severity Impact
Coping with the diseases	Attitude and practice towards changing lifestyle Barriers to accessing health care

### Health and health beliefs

#### Perceptions of health

Men and women had different perceptions of the meaning of ‘good health’. We observed no differences between individuals affected or not affected by POAG, hypertension, and/or diabetes. Men described good health as having a disease-free body, energetic feelings, uninterrupted sleep, eating well with good appetite, feeling comfortable without tiredness, and not having symptoms like pain or swelling:

… I have [high] blood pressure but right now my pressure is well controlled with medicine and I feel better now. Does that mean I am in good health? No, I still feel I have disease so I consider myself unhealthy. (45-year-old man with hypertension)

On the other hand, women described good health as feeling fit for work, ability to undertake household responsibilities, ability to rise on time and perform household chores without any problem, having a light and comfortable body without any pain, and normal appetite. Unlike men, women did not relate good health with absence of disease:

… I consider myself healthy as long as I can do all the household chores and take care of my children. More precisely I believe that as long as I am not bedridden I am healthy. (43-year-old woman with diabetes)

#### Perceptions of causes of disease

Irrespective of being affected or unaffected by disease, both men and women believed that health was their own responsibility and that they suffered from disease because of their unhealthy habits. One man was certain that he got diabetes as a result of unhealthy diet:

… I am very sure that I got this disease of sugar [diabetes] because I ate lots of sugary stuffs in the past. I could never resist when I saw sweets. It is my fault that I got this disease; if I had known earlier, I would have not got this disease. (37-year-old man with diabetes)

Participants also identified external causes as responsible for disease. In particular, men believed that external factors (e.g. air pollution, inorganic fertilizers, lack of regulations in the food market, and lack of proper governmental policies) affected their health. Other men suggested that social and cultural practices negatively influenced their determination to follow recommendations for healthy habits. However, men also expressed the possibility of exercising self-control to change their health behavior:

… In our Nepali culture, during festivals, people eat a lot of fatty fried food and drink alcohol because most often friends and relatives force us to do so. If not, we will feel out of place or will be considered abnormal. I don't say it is completely their fault … maybe we should also have self-control to resist ourselves from doing unhealthy practice. (41-year-old man with hypertension)

Some women believed that health was in God's hands or governed by an evil external power. They stated that some illnesses could only be cured by faith healers (i.e. mediators between the spiritual and the realistic world) or by offering sacrifices to placate the spirit.

### Knowledge of the diseases and their prevention

#### Hypertension and diabetes

Our participants knew that hypertension and diabetes occurred frequently in JD-HDSS, and believed that these diseases resulted from unhealthy diet and lack of physical activity. This belief was similar among men and women and not related to whether or not they were affected by these diseases. Participants perceived that excessive intake of sweets, salt, and fatty food; adulterated cooking oil; excessive alcohol intake; smoking; physical inactivity; and mental stress cause hypertension and diabetes. Additionally, women affected by hypertension and diabetes stated that the diseases were transmitted through their parents:

… I think diseases like diabetes and hypertension come to us through our parents. I think like this because we are seven brothers and sisters, including me. Four of us suffer from diabetes and my father died from diabetes 2 years ago. I am afraid that I may have given it (the disease) to my children as well. (39-year-old woman with diabetes)

This belief led to some differences in opinion. Some argued that a disease transmitted through parents had to be a communicable; others disagreed. Male participants expressed little knowledge about the role of cholesterol in causing hypertension, but some women affected by hypertension and diabetes stated that obesity may increase the risk of most NCDs including hypertension and diabetes:

… I feel that being fat is a disease in itself. I was absolutely fine until I delivered my first child. After this, I started putting on weight and I got this disease of sugar (diabetes). So I feel that excessive body weight invites all diseases. (33-year-old woman with diabetes)

Men and women with hypertension and diabetes described potential consequences as paralysis, loss of speech, kidney failure, sudden death, blindness, delayed wound healing, and infections. The group of women suffering from hypertension and diabetes recalled an incident they had recently witnessed in their community:

… Our neighbor who is just 55 years old, suddenly lost consciousness last week and was rushed to hospital. We have heard that according to doctors he had brain hemorrhage …. but not sure; some were even saying he had very high blood pressure that caused paralysis. Poor man, he is completely bedridden. (Group of women suffering from hypertension and/or diabetes)

In general, men and women without hypertension and diabetes had limited knowledge about these diseases and were unaware of potential complications. They described complications (e.g. stroke, numbness of toes and fingers, and frequent infections), but were not entirely sure whether they were caused by hypertension and diabetes.

#### Glaucoma

Regardless of gender and pre-existing diseases, participants knew that glaucoma was prevalent in JD-HDSS and perceived it as a lifelong, vision-threatening disease. Men with POAG, hypertension, and/or diabetes knew more about glaucoma than other participants. They described POAG as a genetic disease that runs in a family, requires lifelong treatment, may cause blindness, and they had heard about a possible association with hypertension and diabetes. Men also described POAG as a sight-threatening disease:

… My uncle has glaucoma and he is almost blind by one eye. When I went for blood pressure checkup, my doctor said that I may also develop glaucoma. So I need to go to eye doctor. At that time, the doctor also told me that (high) blood pressure and sugar (diabetes) may also be associated with glaucoma. (42-year-old man with hypertension)

None of the female FGD participants suffered from POAG. However, in contrast to male participants, the presence of hypertension and/or diabetes had neither increased women's knowledge about retinopathies that can be caused by these diseases nor provided insight into a possible association of POAG with these diseases. These findings suggest a gender difference in knowledge.

… I don't have glaucoma so I don't know much about it. I have heard that it can cause blindness forever. I am not sure how this disease occurs. Is this similar to cataract? I have seen people getting operated for cataract but not sure whether glaucoma can also be operated. (40-year-old woman with hypertension)

#### Preventive measures

Irrespective of gender and health status, most participants knew that healthy lifestyles and healthy diet can prevent hypertension and diabetes. They described several preventive measures, including being active; doing manual work; walking every day; quitting smoking; avoiding junk food; and reducing the intake of alcohol, salt, sugar, and fatty food. In addition, some women believed that weight loss could prevent hypertension, diabetes, and the complications of these diseases:

… I am sure that if I can reduce my weight, my sugar (diabetes) will get controlled; but reducing weight is so difficult. (33-year-old woman with diabetes)

Some men with hypertension and diabetes knew that high cholesterol might be a risk factor for hypertension, and they knew the need for regular health examinations and blood tests to monitor cholesterol. Other men who were unaffected by disease doubted that these diseases are preventable:

… People often talk about preventing these diseases by doing exercise and manual work. If this was true then why do farmers get these diseases; they work all day on the farm. Their farm work is harder than physical exercise that people often talk about. I think exercise and manual work cannot prevent this disease. (38-year-old healthy man)

Discussions regarding measures to prevent glaucoma were not spontaneous, but rather were initiated by probing questions. Participants were unsure how POAG-related blindness could be prevented. Even those who were aware of a likely association between POAG, hypertension, and/or diabetes, had no knowledge about preventive measures. They knew that early treatment could prevent blindness, but did not understand how to get this treatment or when they should go for an eye examination:

… I am not sure how blindness can be prevented. If I have no problem with my vision why would I go to an eye doctor in the first place? Well, I do not know how this works. I go to my doctor every six months for hypertension, so maybe my doctor will be able to tell me. (42-year-old man with hypertension)

### Perceptions of diseases

#### Severity

Gender and the presence of POAG, hypertension, and/or diabetes influenced participants’ perceptions of disease severity. Men suffering from these conditions perceived them as incurable, life threatening, sight threatening, and dangerous, necessitating lifelong medication:

… Ever since I got this sugar (diabetes) I feel very stressed and worried about my health. For seven months doctors were not able to bring my (blood) sugar down. It is a very difficult disease to treat. But I feel ok now, the blood sugar came to normal just last month and I am very careful with my food these days, I hope it remains under control. (42-year-old man with diabetes)

… Whatever you do, once you get this sugar (diabetes); in my opinion, one cannot live more than ten years. I have seen many dying from sugar. (45-year-old man with diabetes and hypertension)

In contrast to the men suffering from diseases, men unaffected by disease did not regard these diseases as dangerous, but rather described them as controllable with medication. Unlike men, women, regardless of their health status, believed that these diseases are not dangerous in nature. Women and healthy men expressed that people with hypertension and diabetes live longer and have no complications if they take regular medicine (i.e. can live without symptoms with medication). Indeed, some men unaffected by diseases perceived these conditions as curable. Regardless of health status, women perceived POAG as a blinding disease but curable:

… I do not have glaucoma. However, I have heard from people that it can make a person blind forever. But I think it is not a bad disease because I have heard that this can be treated just by eye drops, so this may be a curable disease. (33-year-old woman with diabetes)

#### Impact

Both male and female participants suffering from POAG, hypertension, and/or diabetes consistently mentioned how the diseases affected their lives such as, difficulty coping with dietary restrictions, inability to enjoy their food, increased family stress, increased stress about medication side effects, losing time during hospital visits, reduced working hours and subsequent financial loss, job loss, increased expenses due to treatment and hospital visits, and inability to perform office and farm work with precision due to reduced vision.

We also found gender disparity regarding impact of disease on women's lives. Women with hypertension and/or diabetes expressed that their family members treated them unfairly after diagnosis, and faced more problems than men. They reported that in-laws considered the diagnosis a false claim to avoid doing housework, and expected them to do all the household chores and heavy farm work even when they were not well. Some wives said their husbands did not support them in fighting for their rights and spent little time on their wives health care, even refusing to go with them to hospital. Others said that family members were angry if their food was not ready on time:

… My mother-in-law thinks I am telling lies about being unwell. My husband never supports me and despite of my ill health, I have to cook food for everyone and keep my in-laws happy. I have to rely on my husband's free time to go to hospital and also for money to buy medicine. Life is very difficult for me. (40-year-old woman with diabetes and hypertension)

Men and women without POAG, hypertension, and/or diabetes believed that these diseases could have substantial impact on life but had no in-depth knowledge:

… I suppose, there must be a bigger impact on life for those who suffer from these diseases. I see my uncle going to hospital quite frequently; he has got (high) blood pressure and also some problem in his heart. It must be difficult to cope with all the medicines and hospital visits, but I do not really know what other impacts these diseases can have on life. (39-year-old healthy man)

### Coping with diseases

#### Attitude and practice towards changing lifestyle

Men with hypertension and/or diabetes reported that they tried to change their lifestyle by exercising, walking, and reducing salt and alcohol intake, but their efforts were inconsistent. They recognized that lifestyle changes are not easy but also understood that such changes could improve their lives:

… I know that changing lifestyle is essential for me if I want to live better and have a longer life, so I have no other option than to follow the doctor's advice. I am trying to do exercises and control my diet. At least 4–5 times a week I am doing exercise if not every day. (43-year-old man with diabetes)

Men without disease knew about recommendations concerning food, exercise, tobacco, and alcohol, but felt that it was neither easy to change their lifestyle nor crucial for them to do so. They stated that knowing what to do was easy, but practicing the recommendations was much more demanding. Giving up long-term habits was difficult. Some men did not want to give up good food by reducing salt, sugar, and alcohol intake, but said they would do so in the future if they were diagnosed with the disease, thus indicating a gap between knowledge and practice.

… I agree that practicing healthy habits is good for everyone but how many of us practice healthy habits in real life? Knowing and practicing are two different things. Well, I rather enjoy my life now. I am not ready to give up good food by reducing the intake of salt, sugar, alcohol etc. I will do that when time comes …. I mean if I get diseased in the future. (39-year-old healthy man)

In contrast to men, the presence of disease did not influence women's attitude towards changing lifestyle. Some women even giggled shyly while discussing physical exercise. They mentioned that preparing separate meals meant more work in the kitchen, so they would eat what was cooked for the entire family. They stated that regular exercise or walking was impossible because the amount of work at home left no time for extra activities. Moreover, women expressed reluctance in doing exercise and also said that exercise is not socially acceptable for women. Thus, even if a woman tried to change her lifestyle, her family would not support her:

… Women doing exercise is not acceptable in our society, it is just meant for men. I know we should be doing this, but our family members will never support us. Walking is probably good for us but where is the time? We are tied up in household work so much that we can hardly find free time for ourselves. (35-year-old healthy woman)

… I did try to go for a walk in the morning but my mother-in-law said that this was all drama to avoid the household work, so I stopped. For us (women), maybe household work itself is an exercise, but not sure how much it helps. (30-year-old woman with hypertension)

Some women said that they would have to overcome many barriers, including their children's happiness, if they wanted to take care of their health. One woman wondered if she should change her lifestyle by ignoring her family's happiness:

… Forget about in-laws; if I start taking a one-hour walk every day, even my children will not be happy with me. Everyone at home expects me to have their food, clothes, tea, and snacks ready for them in time, and if I start taking care of myself I will be late for all these household chores. If I really want to change my lifestyle, I will have to ignore the happiness of my family. I am not sure whether I should do this for my health. (44-year-old woman with diabetes)

#### Barriers to accessing health care

Although both male and female participants discussed several barriers to accessing health care, including lack of knowledge, expensive health care, lack of funds, and a long waiting time at the hospital, women faced several more barriers. Although barriers differed between men and women, none were influenced by presence of POAG, hypertension, and/or diabetes (Table 2).

… I know people who suffered from cancer or kidney failure and sold their property like house and land for treatment, but in the end had no life and no money. (28-year-old healthy man)

**Table 2:** Framework chart of barriers to accessing health care

Barriers to accessing health care	Men with disease	Healthy men	Women with disease	Healthy women
Educational/information	Lack of knowledge about disease No information about hospitals and doctors	Lack of knowledge about disease Lack of education	Lack of knowledge about disease Lack of education Lack of confidence to travel alone	Lack of knowledge about disease Lack of education
Institutional	Crowded hospitals and difficulty finding a doctor Long waiting time in hospital	Hospital not good Long waiting time in hospital	Long waiting time in hospital Doctors not available in the hospital	Long waiting time in hospital
Economic	Healthcare expensive Lack of money	Healthcare expensive	Healthcare expensive Lack of money	Healthcare expensive Lack of money
Cultural			No family support Lack of independence to spend money Lack of decision-making power Dependent on family	Lack of independence to spend money Lack of decision-making power
Behavioral	Casual attitude	Lack of time	Lack of time	Lack of time

Some men said that people exhibit a casual attitude towards health and seeking health care. Others mentioned the lack of proper information regarding where to go for proper health care or who was the best physician for a particular disease. Table 2 illustrates various barriers faced by men and women. In contrast to men, women reported several obstacles in accessing health care that exists in Nepalese society including inadequate education and health awareness and lack of decision-making power, self-confidence, and independence to spend money.

… Nothing is in women's hands. Since we do not contribute to household earnings we cannot spend as we like. To make it worse, I cannot even travel alone to hospital for checkups. My mother in-law decides whether I should go or not, and my husband takes me to hospital. (40-year-old woman with diabetes)

## Discussion

There is an ongoing debate regarding the possible association of POAG with hypertension and diabetes. Although some studies reveal such association (4–6) , others refute that finding (24, 25). However, more recent reports (26–28) , including our own recently published study (7), demonstrate that POAG may associate with hypertension and diabetes.

Earlier reports from Nepal predicted a gap in knowledge regarding glaucoma among the patients attending the hospital and possible barriers to seeking health care (7, 13). Furthermore, as urbanization enhances the prevalence of hypertension and diabetes (29, 30), there is an increasing probability of emergence of POAG in the community. Thus, the present study aimed to explore the knowledge of POAG together with hypertension and diabetes and health-seeking behavior in JD-HDSS, a peri-urban community in Nepal.

This study was conceptualized on the basis of both health-related behavior and health theories. We used the HBM as a main concept along with health literacy and health locus of control. The theory of HBM hypothesizes that health behavior of an individual depends on four dimensions of beliefs (14). They are: subjective perception of being at risk of disease; perception of severity of disease in terms of medical or social consequences; beliefs regarding the benefit of an action that is known to reduce the disease threat or complication and; the perception of barriers against the action. These barriers may be in the form of expensiveness, dangerous outcome, unpleasantly painful or difficult, time-consuming, and so on. Therefore, for an individual to take action for good health, the benefits should outweigh the barriers.

Anticipating the cultural norms, we thought women would face difficulty in expressing their views in front of men (31). Thus we conducted separate FGDs for men and women. Globally, NCDs accounted for 9 million premature deaths occurring before 60 years of age (32). This suggests that NCDs begin early in life, before clinical manifestations appear, which is also reflected in earlier reports from Nepal (9, 10) that demonstrate hypertension and diabetes in younger age groups, i.e. individuals <40 years of age. This further justified our decision to enroll younger participants in our study, in addition to the fact that the Nepal Demographics Profile of 2013 (33) shows the highest proportion of the Nepalese population belonging to the age group 25–45 years. This furthermore includes the most active people. Work and employment maximize their opportunity for exposure to the outer world and increase the likelihood that they will gain more knowledge than other groups in the community.

### Understanding about general health

Perceived meaning of good health differed among male and female participants. Connecting good health with women's ability to undertake household responsibility mirrors the results of a study from Scotland, which reports that women prioritize their family and household over their own health (34). This indicates women's universal nature of considering their health less important than fulfilling their family and household responsibilities. As per explanation given by HBM (14), women in this community are less likely to change their health behavior, particularly regarding self-care and preventive measures.

Our study demonstrated mixed opinions regarding health locus of control, regardless of gender. In contrast, a study from Israel reported a predominance of external health locus of control among women (35), possibly due to variations in culture, age of study participants, and/or data collection methods. The Israeli study enrolled participants aged 50–75 years and collected data by telephone interviews, compared to 25–45-year-old participants and FGD data collection in our study.

Individuals with internal health locus of control believe that their own behavior and active involvement in health care contributes importantly to improved health outcomes (15). Thus, the community might learn to take an active role in self-care of NCDs. Apart from environmental, social, and cultural factors, some women believed that God or a higher power might control their health. Thus, the human tendency to blame others for threatening events might explain the external health locus of control (36). A study from Poland reports similar mixed beliefs of health locus of control in patients with chronic illnesses (37).

### Knowledge of hypertension, diabetes, and POAG

In contrast to a study from a rural community in Pakistan that demonstrates poor awareness about diseases (38), our participants were aware of a rising prevalence of hypertension and diabetes in the community, and believed this was related to inactive lifestyle and unhealthy diet. Our female participants perceived obesity as a risk factor for hypertension and diabetes, whereas men did not talk about obesity. Similarly, men in Tanzania underestimate their body weight and do not perceive obesity as a health threat (39), whereas Tanzanian women are more conscious of their appearance/bodies (40).

In our study, both male and female participants who suffered from hypertension and diabetes showed greater understanding of the consequences, compared to healthy participants, possibly due to curiosity about their own disease and its complications. However, greater knowledge could associate with frequent meetings with doctors and encounters with other people suffering from similar diseases, or indicate a positive reflection of health information received from the hospital. Indeed, a study from Malaysia also demonstrates that people with pre-existing diseases know more about the potential consequences of their disease (41).

Regardless of gender and pre-existing disease, our participants had heard about glaucoma and were aware that glaucoma was prevalent in JD-HDSS. In contrast, studies from India and Ethiopia report that most people have poor awareness of glaucoma in the community (42, 43), possibly due to differences in study design as both above studies conducted face-to-face interview in contrast to our FGDs. Additionally, in contrast to our peri-urban study site, outside the capital city of Kathmandu in Nepal, studies from India and Ethiopia were conducted in rural areas. Men suffering from POAG, hypertension, and/or diabetes and those who had a family history of glaucoma in our study exhibited more knowledge about POAG. Similarly, a study from India (44) demonstrates correlation between knowledge of disease, family history of glaucoma, and motivation to seek information. None of the women in our study had pre-existing glaucoma but they suffered from hypertension and/or diabetes. Despite these diseases, women did not demonstrate knowledge regarding the effect of hypertension and diabetes on eyes, or in-depth knowledge regarding glaucoma in particular. This suggests a gender disparity in health literacy relating to glaucoma and is consistent with a policy brief published by Nepal Gender and Eye Health Group that reports gender inequity in health care and a gap in health literacy among women compared to men (45).

Poor knowledge among healthy individuals indicates that in JD-HDSS, health literacy is generally poor (16). More importantly, limited health literacy in healthy individuals may lead to higher risk of non-response to health education and failure to follow health recommendations, further increasing the prevalence of NCDs.

Unsurprisingly, participants affected by disease knew more about causal factors, consequences, and preventive measures. However, it is clear that knowledge among participants already affected by disease can only prevent further complications. A study from Malaysia also reveals that greater knowledge among healthy people could result in active community participation in promoting preventive measures leading to reduced prevalence of disease (41). Although our participants had knowledge of general aspects of preventive measures for hypertension and diabetes (e.g. healthy diet and active lifestyle), more specific and focused health education about NCD prevention will be required to bring changes in people's health behavior. The present study revealed lack of knowledge related to preventive measures for POAG, suggesting the need for a health awareness program focused on reducing the prevalence of glaucoma blindness.

### Addressing the disease and its impact on life

Men affected by POAG, hypertension, and/or diabetes perceived these diseases as serious, incurable, and dangerous. In contrast, women, regardless of status of their health, and healthy men without disease did not share this perception; some even believed these diseases might be curable. Similar to our findings, non-diabetic men and women attending a primary health care center in Karachi, Pakistan, show poor awareness of diabetes and its consequences and believe that diabetes is not a serious disease (46). A study in India also demonstrates that women exhibit poor awareness regarding diabetes and perceive themselves to be at low disease risk (47). Additionally, a study from United States also demonstrates that women perceive these health risks lower than the actual risk (48) indicating that women, regardless of where they live, unanimously appear to perceive a lower health risk which may make them more vulnerable towards disease. According to the HBM, healthy men and women in JD-HDSS may be at higher risk of developing NCDs because they do not perceive them as serious and therefore are less likely to take preventive action (14). It is worrisome that there is gross gender disparity regarding health issues in our community. Because women with hypertension and diabetes believed that these diseases were not serious despite the knowledge of complications, there is greater risk of developing complications in them. Women in this community may remain ignorant and abstain from adequate self-care and preventive measures, which in turn may prove detrimental to their health.

Both men and women suffering from POAG, hypertension, and/or diabetes described the impact of disease on their daily lives and how they faced difficulties in balancing their family life and financial status. They expressed that these difficulties were due to several reasons such as dietary restrictions resulting in increased workload in the kitchen, increased stress of disease within the family, job loss with subsequent financial loss, increased expenses due to treatment, and hospital visits. This finding is similar to recently published report from the JD-HDSS community (49). In addition to such common difficulties expressed by both genders, women who suffered from hypertension and/or diabetes reported additional negative impact on life. They expressed that they lacked support and understanding from family members indicating a gender bias in understanding the health of women in the family. Another study from Nepal found that women with chronic diseases received inadequate family support and faced negative attitudes from family members (50), likely causing difficulty in managing chronic diseases and possibly increasing morbidity and mortality (51, 52).

### Attitude and practice: changing lifestyle for better health

In accordance with their knowledge about risk factors, men with hypertension and/or diabetes expressed willingness to change their lifestyle, suggesting the relationship between the knowledge and health attitude that reflects the hypothesis of HBM (14). Similarly, a study from Sweden showed a positive correlation between knowledge about risk factors for coronary heart disease and lifestyle changes (53). Despite knowledge about health recommendations relating to food, exercise, tobacco, and alcohol, healthy men from JD-HDSS were unwilling to change their lifestyle because they doubted the benefits and were not sure about their susceptibility to disease. Such behavior reflects a negative attitude towards health because they did not believe that their action would benefit their health as explained by the HBM (14). This indicates a gap between knowledge, attitude, and practice. Women were reluctant to change their lifestyles, regardless of health status, even though they had knowledge regarding disease consequences and impact on life. Such reluctance seems to be related mainly to perceived social and cultural barriers, including lack of support from spouse and family. Previous studies originating from the United States and Nepal report that women feel lack of spousal and family support for changing life style (54, 55). Thus, our study suggests that there is a gap between knowledge, attitude, and practice among women in relation to health care.

### Accessing health care

Healthy participants and those who suffered from POAG, hypertension, and/or diabetes analogously perceived barriers to seeking health care. In addition to educational, institutional, and economic barriers commonly described by both men and women, women expressed several barriers that are pertinent to cultural norms. A previous study from Nepal reports a similar lack of decision-making power, which makes women unable to access health care services without permission from family members (55). Nepal Gender and Eye Health Group has published a policy brief (45) that reports underutilization of health care services by women as compared to their men counterparts and also describes the cultural barriers faced by women. A study from the United States reports similar gender disparity in the perception of barriers to health care in low-income countries (56). The present study suggests that gender inequalities limit access to health care in JD-HDSS. Indeed, the barriers women face in accessing health care and adopting preventive measures for better health outcomes suggest a significant need for tailored programs.

### Strengths and limitations

Our FGDs included a relatively large (for a qualitative design) and representative sample of participants. Prior to data collection we tested the FGD guide in a group of eight participants from the same population. The moderator discouraged participants to ask and discuss any health-related question before, during, and after each FGD to ensure that participants’ knowledge about disease is not affected by the prior discussion with researcher. Repetition of responses from one FGD to another was considered as saturation point. Codes, categories, and themes represent the consensus of both authors.

Because this study was conducted in the JD-HDSS, which is the subject of regular surveys, the community's knowledge about diseases may be higher than normal. Consequently, our findings may not accurately represent the health literacy of Nepal's general population. Moreover, although educational status is known to influence knowledge of disease and willingness to adopt lifestyle changes, we did not consider information about participants’ educational status for FGD.

## Conclusion

The present study reveals understanding about POAG, hypertension, and diabetes from the perspective of people living in the JD-HDSS, a peri-urban community in Nepal. Individuals with pre-existing diseases exhibited adequate knowledge about hypertension and diabetes, but there was a gap in knowledge regarding POAG. We demonstrated gender disparity in terms of glaucoma health literacy, perception of disease, and access to health care, suggesting that women may be more vulnerable to disease and its complications. We also found a gap between knowledge, attitude, and practice in health in women and healthy men. Nevertheless, because people in JD-HDSS, including women, showed internal health locus of control, tailored population-based health education programs with more emphasis towards women may increase awareness regarding the importance of lifestyle change and the need for self-care.
